# Probiotic Potential and Technological Properties of Bacteriocinogenic *Lactococcus lactis* Subsp. *Lactis* UTNGt28 from a Native Amazonian Fruit as a Yogurt Starter Culture

**DOI:** 10.3390/microorganisms8050733

**Published:** 2020-05-14

**Authors:** Gabriela N. Tenea, Jimena Suárez

**Affiliations:** Biofood and Nutraceutics Research and Development Group, Faculty of Engineering in Agricultural and Environmental Sciences, Technical University of the North, Av. 17 de Julio s-21 Barrio El Olivo, Ibarra 100150, Ecuador; jgsuarezi@utn.edu.ec

**Keywords:** *Lactococcus lactis*, *Streptococcus thermophilus*, probiotic, protein, starter culture

## Abstract

A native *Lactococcus lactis* subsp. *lactis* UTNGt28 (GenBank accession no: MG675576.1) isolated from Amazonian fruit of the tropical Caimitillo (*Chrysophyllum oliviforme*) tree and the commercial strain *Lactococcus lactis* subsp *lactis* ATCC11454 (LacAT) were targeted ex vitro in whole milk in combination with *Streptococcus thermophilus* ATCC19258 to obtain a fermented probiotic beverage. Concomitant with cell viability determination during storage (28 days), the pH, titratable acidity, syneresis, protein and fat were evaluated. The results indicated that neither UTNGt28 nor LacAT displayed a high capacity to ferment whole milk and survive during storage; a statistically significant difference (*p* < 0.05) in cell viability was registered for UTNGt28 compared with LacAT when inoculated alone or in combination with *S. thermophilus.* A principal component analysis showed a clear difference between the yogurt formulations at day 1 and 28 of storage. The PC 1 explained 46.8% of the total variance (day 28), was loaded in the negative (−) direction with titratable acidity (% lactic acid), while the PC 2 explained 22.5% (day 1) with pH. PC 1 was loaded in the positive (+) direction with pH, cell viability, syneresis, fat and protein. Overall results indicated that UTNGt28 has the technological properties for further development of a new probiotic product.

## 1. Introduction

Historically, lactic acid bacteria (LAB) have been used for food production, as they improve flavor, aroma and texture characteristics, complying with consumer needs. LAB strains as a starter culture produce metabolites with an overall inhibitory capacity, contributing to the extension of the shelf life of food products [1]. Currently, some LAB strains are known as probiotics, that, when administered in adequate amounts, confer a health benefit on the host [2]. Most probiotic bacteria belong to the genera *Lactobacillus* sp. *Bifidobacterium* sp., *Lactococcus* sp., and some to *Bacillus* sp., and have been detected in various environments [3,4]. The starter bacteria *Streptococcus thermophilus* is known for its use in the production of yogurt, as it is the only species from its genera recognized as Generally Recognized As Safe (GRAS) by the Federation of Drug Administration [5,6]. *Lactococcus lactis* was primarily isolated from fermented raw milk and kefir [7], also a GRAS strain, was characterized with respect to their physiology and molecular genetics [8]. Its fundamental function during milk fermentation is the rapid conversion of lactose into lactic acid, preventing the growth of pathogens in the fermented product [9]. In addition, due to its proteolytic activity and the conversion of amino acids, it contributes to the final texture (moisture, softness) and taste of dairy products.

Although most probiotic species are of human origin, there are several studies presenting scientific evidence of the probiotic potential of LAB strains isolated from unconventional sources such fruits, vegetables, juices, and grain products [10,11,12]. Two species of *Lactobacillus* genera were isolated from the fermentation of cocoa (*Theobroma cacao* L.) and showed protective effects against *Salmonella typhimurium* [13]. Thus, it is of interest to extend the probiotic microorganisms “list”, adding bacteria from spontaneously fermented foods. Those microorganisms constitute the microflora of an microecosystem in which they were produced, thus, if tested in terms of safety, they may constitute an interesting alternative to gut bacteria as they might possess healthy properties, beyond their technological functions [14].

Probiotics’ product development and the promotion of probiotic consumption has been successful in Europe and Asia, and, more recently, in other regions of the world. In Ecuador, the milk industry is using commercial strains to produce yogurt and cheese. The market is limited to a few registered trademark products containing patented strains such as *Lactobacillus rhamnosus* GG (ATCC53100) [15]. In light of extensive changes made by the governmental policy of Ecuador, several undeveloped natural areas, such as subtropical native forests (reservoirs of medicinal plants and fruits) were considered as important genetic resources to be exploited for biotechnological research. However, the bacterial microbiota of wild-type fruits originating from the Amazonian rainforest of Ecuador were investigated in order to identify and characterize new LAB species [16]. Thus, several isolated strains from the genera *Lactobacillus, Lactococcus* and *Weissella*, showed a high capacity to tolerate bile salt at a physiological concentration and high acidic conditions, as well as antimicrobial capacity in vitro [16]. It is likely that the microorganisms from this environment could bring a new source of functional compounds; they might have better viability in the food matrix and improved organoleptic characteristics as well as functional properties (antioxidant capacity), thus demonstrating technological and functional properties that could replace the commercial strains originated from the human intestine, supplying similar or improved characteristics. Among them, *Lactococcus lactis* subsp. *lactis* UTNGt28 exhibits a high capacity to inhibit several food-borne pathogens [17]. In this study, the probiotic properties of the UTNGt28 strain was evaluated in whole raw milk alone or in combination (at different doses) with a commercial starter culture *Streptococcus thermophilus* ATCC19258 to obtain a fermented milk or yogurt formulation with probiotic capacity. In addition, the results were compared with the yogurt formulations obtained by combining the commercial strain *Lactococcus lactis* subsp. *lactis* ATCC11454 in different doses with *Streptococcus thermophilus* ATCC19258. The combination ratio of the bacterial culture and the strains might influence the quality of the final product. 

## 2. Materials and Methods 

### 2.1. Microorganisms and Growth Conditions

*Lactococcus lactis* subsp. *lactis* UTNGt28 (GenBank accession no. MG675576.1) was isolated from Amazonian wild immature fruit of the tropical Caimitillo (*Chrysophyllum oliviforme*) tree [17]. The commercial strain *Lactococcus lactis* subsp. *lactis* ATCC11454 and *Streptococcus thermophilus* ATCC19258 were used for comparison and starter culture, respectively. The strains were maintained as frozen stock cultures in MRS broth (Difco, Detroit, MI, USA) containing 20% (*v/v*) glycerol.

### 2.2. Yogurt Formulations Manufacturing

#### 2.2.1. Cell Inoculum 

The LAB strains were activated and routinely sub-cultured in MRS broth under anaerobic conditions at 37 °C. Nine cell cultures were prepared as follows: a) UTNGt28 + *S. thermophilus* ATCC19258: 1:1 (*g/g*) (T1); b) UTNGt28 + *S. thermophilus* ATCC19258: 1:3 (*g/g*) (T2); c) UTNGt28 + *S. thermophilus* ATCC19258: 3:1 (*g/g*) (T3); d) UTNGt28 alone (T4) e) LacAT + *S. thermophilus* ATCC19258: 1:1 (*g/g*) (T5); f) LacAT+ *S. thermophilus* ATCC19258: 1:3 (*g/g*) (T6); g) LacAT+ *S. thermophilus* ATCC19258: 3:1 (*g/g*) (T7); h) LacAT alone (T8); i) *S. thermophilus* ATCC19258 alone (T9).

#### 2.2.2. Raw Milk

Raw milk was purchased from the Santa Monica University farm, Imbabura, located in northern Ecuador (cattle breed Holstein). The milk was used pasteurized for 12 min at 100 °C to provide lactic acid bacteria-free milk [4]. The milk quality was evaluated for acidity, fat, relative density and total protein prior to inoculation. 

#### 2.2.3. Inoculation of Cell Mixture in Milk

The bacterial precultures (5 mL) were transferred into flasks of 100 mL MRS broth and incubated for 18 h at 37 °C to obtain the inoculum biomass; the cells were harvested by centrifugation (5000× rpm for 10 min at 4 °C) and washed once with sterilized 0.1 M sodium phosphate buffer at pH 7.0. Each bacterial culture mixture as described above was inoculated in 750 mL pasteurized whole milk. The inoculated mixtures were distributed in glass jars, five jars per culture combination, then incubated at 42 °C until a pH of 4.5 was reached. The fermentation time was registered for each combination. All bottles were cooled to 5 °C for 4 h, and 4% sterile glucose was added gently by manually mixing the bottles under the sterile flow-chamber; the end of this procedure was considered day 0. The bottles were stored in a refrigerator for 28 days; starting with day 1 of storage, every 7 days one bottle per combination (150 mL) was taken to determine the titratable acidity, pH, syneresis, and viability of the inoculated bacteria. The study was conducted in triplicate, starting with a new batch of milk. In addition, using a similar approach, the fermentation was performed at 35 °C for the UTNGt28 formulation alone (T4) and the pH and acidity were monitored and compared with the formulation obtained at 42 °C.

### 2.3. Evaluation of Physicochemical Properties of Yogurt Formulations

pH was measured by electrode immersion with a pH meter (S210, Mettler Toledo, Columbus, OH, USA). The percentage of titratable acidity was determined by titration of 10 g of yogurt with 0.1 N NaOH using phenolphthalein as the indicator [18]. Results were expressed as grams of lactic acid per 100 g of yogurt product. These determinations were performed in triplicate. To evaluate syneresis, 20 g of fermented milk was taken and poured onto the Whatman filter paper (Grade 1, Sigma-Aldrich Co. LLC, Saint Louis, MO, USA) placed on top of a funnel. The amount of filtrate collected after 1.5 h of drainage was the index of the water retention capacity of fermented milk. The syneresis (%) was calculated according to the following equation: Syneresis (%) = (amount of filtered whey (mL))/(20 g of sample) * 100%. All measurements were performed at day 1, 7, 14, 21 and 28 of storage. Similarly, the syneresis was monitored during storage in the UTNGt28 formulation obtained at 35 °C. 

### 2.4. Determination of Cell Viability during Storage

The cell population of yogurt starters was determined at day 1, 7, 14, 21 and 28 of storage. One gram of yogurt formulation obtained was diluted in 9 mL of sterile peptone (Merck, Darmstadt, Germany) water (0.1%), and appropriate dilutions were plated on MRS agar (Difco, Detroit, MI, USA) for enumeration of total lactic acid bacteria in each yogurt formulation [19]. Colony-forming units (CFU) per g of yogurt were recorded for plates containing 20 to 350 colonies.

### 2.5. Determination of Total Protein and Fat in the Yogurt Formulations

The total protein of the crude milk and yogurt formulations was analyzed using the Kjeldahl method [20]. Digestion and distillation were performed with a Kjeltec Auto 1030 Analyzer (Tecator, Höganäs, Sweden). The Gerber method was used as the reference for milk fat content, and total solids were determined by oven drying [21]. All measurements were made in triplicate.

### 2.6. Bacteriological Quality Analysis of the Obtained Yogurt Formulations

Bacteriological analysis (total coliforms, presence of *Salmonella E.coli* and fungi and yeasts) was performed using the plate count agar method to ensure that no harmful microorganisms were present in the milk before and after inoculation with probiotic bacterium mixture [22].

### 2.7. Statistical Analysis

The results were reported as mean ± standard deviation. The normal distribution of the data was employed with Shapiro–Wilk test (RStudio v. 1.2.1335, RStudio, Inc., Boston, MA, USA, 2019). One-way ANOVA and Tukey’s means comparison test were performed to determine significant differences (*p* < 0.05) in the pH, acidity and viability results (SPSS 13.0, Inc., Chicago, Il, USA). A principal component analysis (PCA) was conducted on the cell counts, pH, acidity, syneresis fat and protein determinations using the correlation matrix at day 1 and 28 of storage. The effect of temperature on cell viability during storage was evaluated using ANOVA with split-plot experimental design. Then, Duncan’s multiples range tests and Least Significant Difference (LSD) with Bonferroni correction were applied to determine significant differences between the means.

## 3. Results and Discussion

### 3.1. Milk Quality 

The physicochemical quality of fermented milk products is influenced by the chemical composition of milk, processing conditions, starter culture type and the final pH during cold storage [4]. The fatty acid profile and relative protein content of different starter cultures are the key variables used to identify the effect of bacteria on the flavor and aroma characteristics of the final product. Milk is a suitable environment for growing lactic acid bacteria, thus it is important to evaluate its properties considering that the new candidate, UTNGt28 strain, was isolated from a fruit microenvironment. The physicochemical characteristics of the milk used in this study are indicated in Table 1. The milk characteristics of this study comply with the established parameters of the Ecuadorian standard [23]. Bacteriological analysis indicated that the whole raw milk was free of any contaminant (data not shown), thus was suitable for further use.

### 3.2. Physicochemical Properties of Probiotic Yogurt Formulations

#### 3.2.1. Gel Forming

The consumption of fermented dairy products has increased [24]. Concomitantly, it translates into a considerable improvement in milk utilization and the intake of valuable nutrients [25]. The formation of gel matrix via the aggregation of casein micelles arises when the pH approaches 4.5–4.6 as a result of lactic acid production during fermentation [26]. In this study, the fermentation times varied with the bacterial combination ratio; the shortest fermentation time was registered for T4 and T9 formulations, while the highest fermentation was registered for T8 (Table 2). The differences in fermentation time might be attributed to the capacity of each bacteria to adapt, grow and ferment the milk matrix. A delay in gel formation was observed when the probiotic bacterium dose was above the *S. thermophilus* dose, indicating a possible inhibition of the starter culture by the probiotic bacteria. Previous research indicated that probiotic *L*. *casei* slows the growth of *S. thermophilus* and *L*. *delbrueckii* ssp. *bulgaricus* in milk [27].

#### 3.2.2. pH and Titratable Acidity during Storage

**Figure 1:**
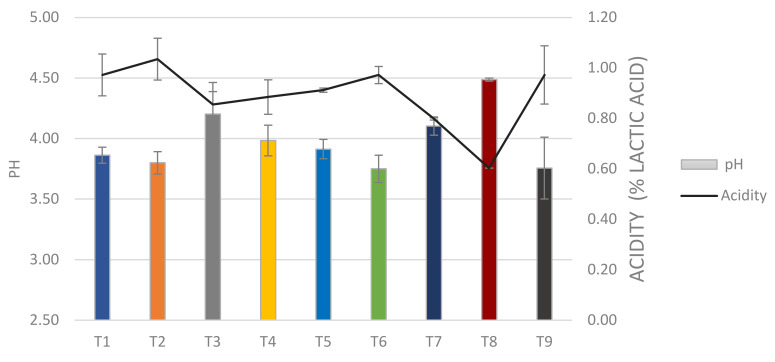
Variation of pH and acidity of the yogurt formulations at day 28 of storage. Legend: T1: UTNGt28 + *S. thermophilus* ATCC19258:1:1 (*g/g*); T2: UTNGt28 + *S. thermophilus* ATCC19258:1:3 (*g/g*); T3: UTNGt28 + *S. thermophilus* ATCC19258:3:1 (*g/g*); T4: UTNGt28; T5: LacAT+ *S. thermophilus* ATCC19258:1:1 (*g/g*); T6: LacAT+ *S. thermophilus* ATCC19258:1:3 (*g/g*); T7: LacAT+ *S. thermophilus* ATCC19258:3:1 (*g/g*); T8: LacAT; T9: *S. thermophilus* ATCC19258. Bars represent the pH and the solid line is % of lactic acid.

The pH values of yogurt formulation in this study decreased during storage due to the residual activity of the bacterium (Figure 1). The lowest pH values of 3.75 and 3.76 were registered for T6 and T9, respectively, at 28 day of storage; T8 presented less acidification (pH 4.49) while the pH of the other combinations declined considerably during storage. The changes in the pH were monitored during storage in all the yogurt formulations (Supplementary Figure S1). The yogurt formulations obtained with the UTNGt28 strain were comparable with LacAT, which also showed a tendency of pH to decrease with increased concentrations of *S. thermophilus* cells. ANOVA analysis revealed that yogurt formulation and storage times had a significant effect (*p* < 0.001) on the production of lactic acid. Our results agree with previous reports, corroborating the residual acidification from day 1 to the end of storage [28]. The acidity values at day 1 of storage varied from 0.65 to 0.84% in the UTNGt28-based formulations and from 0.52 to 0.78% in LacAT-based formulations, indicating that the post-acidification of yogurt depends on the LAB combination used for the yogurt fermentation. At the end of storage, the acidity varied from 0.86 to 1.04% in the UTNGt28-based formulations and 0.60 to 0.97% in the LacAT-based formulations; the T8 formulation remained the least acidic (Supplementary Figure S2). According with the Codex standard for fermented milks [29], yogurt is strictly defined as milk fermented with symbiotic starter cultures of *S. thermophilus* in a viable state, active and still present in the product through the end of shelf life; a minimum of 0.3% lactic acid corresponds to fermented milk, while a minimum of 0.6% lactic corresponds to yogurt. In this study, the acidity values vary with the formulation, however, the products obtained were classified based on acidity as yogurt supplemented with probiotic bacteria. Previous research indicated that some probiotic strains have poor acidification performance in milk compared with common yogurt starter culture [27]. From the industrial point of view, acidification is an important criterion to select the appropriate yogurt formulation. Although a complete sensorial analysis was not conducted, a difference in taste and flavor were observed between the nine obtained formulations. A trained panel of university personnel (15 persons) tasted the yogurt samples during storage, and showed preferences for T1, T3, T4 and T7. The T4 formulation displayed a fruit flavor, suggesting that the microorganism’s origin might contribute to the improvement in the organoleptic properties of the final product, but further investigation is required to identify the volatile compounds that might have a significant influence on the final product flavor.

#### 3.2.3. Syneresis

Serum release is considered an important parameter for the quality of yogurt during storage [4]. The presence of solids in milk determines the increase in interactions between the proteins that constitute the three-dimensional nature and the compaction of the microstructure of the gel [4]. A study by Fiorentini and colleagues [30] indicated that the syneresis drops due to the combination of probiotic strain with *S. thermophilus*. The values of syneresis, represented as percentage of detached water of the yogurt formulations, are indicated in Table 3. A reduction in syneresis was observed from day 1 to 28 of storage; the lowest value was registered for the T2 combination, corresponding to the UTNGt28 and *S. thermophilus* (1:3), T6 and T9, corresponding to the combination LacAT: *S. thermophilus* (1:3) and *S. thermophilus* alone, suggesting that the decrease in the values of detached water is related to a greater amount of *S. thermophilus* in the yogurt formulation. The yogurt formulation obtained with the LacAT only (T8) or in combination with *S. thermophilus* showed comparable values as obtained with UTNGt28. It is known that during yogurt preparation, a stabilizer or gum is added to favor the retention of water because they contribute to the mesh effect in the three-dimensional network of the gel formed [31]. In this study, no stabilizer was applied, thus, during storage the syneresis values declined, with the lowest value registered for the T6 and T9 formulations at 28 day of storage. This drop in syneresis correlates with the drop in pH as both formulations register the lowest pH value at the end of storage (Figure 1). The values of syneresis were lower when use a probiotic strain, however, values >40% were registered when probiotic *L. delbrueckii* was combined with *S. thermophilus* due to the absence of solids [30]. Considering the variable syneresis as an indicator of the quality and stability of the product, the best combinations selected were: T1, T3, T4, T5, T7 and T8. If the stability of lactic acid produced during storage is considered as the principal criteria for the most advantageous formulations, the T1, T2 and T6 were the most satisfactory. Using a similar approach, but with the fermentation temperature at 35 °C, the gel forming time diminished by 7 h, the pH and acidity values were nearly constant with the results obtained when incubated at 42 °C. The acidity value registered at 28 day of storage was 0.94% in the UTNGt28 formulation obtained after fermentation at 35 °C, while at 42 °C it was 0.89%. On the other hand, syneresis dropped to 31% at day 28 of storage, indicating that increasing the temperature enhanced the syneresis (Supplementary Figure S3). Although a mesophilic strain, UTNGt28 showed the capacity to ferment at 42 °C without the need for *S. thermophilus* and the fermentation resulted in a product with favorable properties.

### 3.3. Cell Viability Determination during Storage in the Yogurt Formulations

From a technological standpoint, maintaining cell viability during storage in yogurt formulations supplemented with probiotic cultures is not an easy task, as the viability might be influenced by different factors, such as acid accumulation, strain variation, and interaction of probiotic strain with the starter culture [32]. Research reports indicate that some commercially available dairy products contain an insufficient number of viable probiotics (below <10^6^ CFU/g before the expiration date), thereby diminishing the potential health benefits conferred by these products [33].

**Figure 2:**
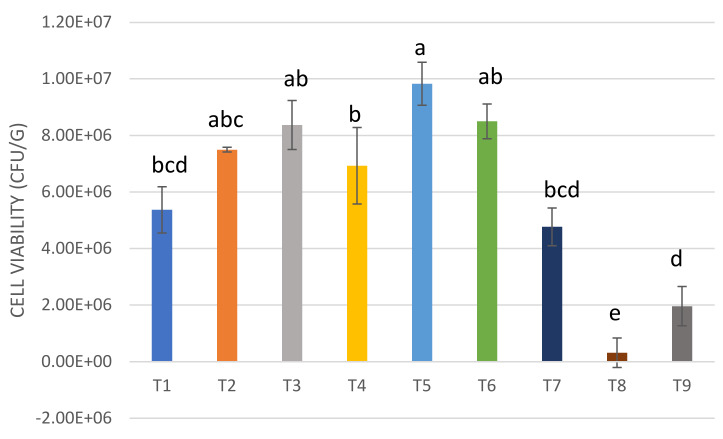
Comparison between cell counts of yogurt formulations at Day 28 of storage with refrigeration. The experimental values (means and standard deviations for *n* = 3) that have no common superscript are significantly different (*p* < 0.05); where a is the highest viability and e the lowest viability. Legend: T1: UTNGt28 + *S. thermophilus* ATCC19258:1:1 (*g/g*); T2: UTNGt28 + *S. thermophilus* ATCC19258:1:3 (*g/g*); T3: UTNGt28 + *S. thermophilus* ATCC19258:3:1 (*g/g*); T4: UTNGt28; T5: LacAT+ *S. thermophilus* ATCC19258:1:1 (*g/g*); T6: LacAT+ *S. thermophilus* ATCC19258:1:3 (*g/g*); T7: LacAT+ *S. thermophilus* ATCC19258:3:1 (*g/g*); T8: LacAT; T9: *S. thermophilus* ATCC19258.

Changes in the viable cell counts (CFU/g) of native and commercial strains in the yogurt formulations during cold storage (4 °C) was monitored. The results indicated a statistically significant difference in the viability (*p* < 0.05) between the native and commercial strains, with T8 registering the lowest viability at day 28 of storage (Figure 2).

**Figure 3:**
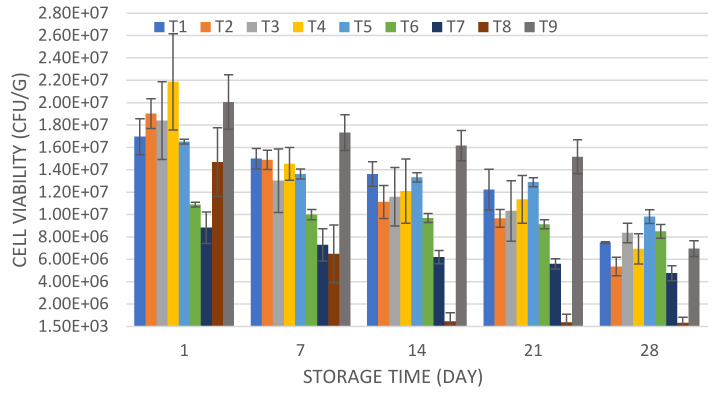
Cell viability (CFU/g) during storage: A) UTNGt28-based formulations; B) LacAT-based formulations. Legend: T1: UTNGt28 + *S. thermophilus* ATCC19258:1:1 (*g/g*); T2: UTNGt28 + *S. thermophilus* ATCC19258:1:3 (*g/g*); T3: UTNGt28 + *S. thermophilus* ATCC19258:3:1 (*g/g*); T4: UTNGt28; T5: LacAT+ *S. thermophilus* ATCC19258:1:1 (*g/g*); T6: LacAT+ *S. thermophilus* ATCC19258:1:3 (*g/g*); T7: LacAT+ *S. thermophilus* ATCC19258:3:1 (*g/g*); T8: LacAT; T9: *S. thermophilus* ATCC19258. The values represent the means and standard deviations for *n* = 3.

It has been suggested that probiotics should be detected in food products at a minimum amount of 10^6^ CFU/g, to compensate for the loss of cells during the passage through the upper and lower parts of the gastrointestinal tract [34]. As determined, the cell counts in the UTNGt28-based formulation fulfilled the requirements for being considered a probiotic bacterium, as the values were above the threshold limit of 1 × 10^6^ CFU/g [35]. Based on our results, by combining the *S. thermophilus* with UTNGt28 in different proportions, the overall cell viability increased, while UTNGt28 counts alone were superior to *S. thermophilus* counts, indicating that UTNGt28 does not need the commercial strain to maintain its viability (Figure 3).

Early research indicated that *S. thermophilus* in symbiosis with another lactic acid bacteria promotes the growth of the attached culture [36]. The viability of LacAT alone (T8) was below the threshold (3.2 × 10^5^ CFU/g), indicating that this strain does not fulfill the normative requirements of the viability of a probiotic strain [35]. Although the T8 formulation showed the highest pH value and lowest acidity at the end of storage, the viability was significantly lower (*p* < 0.05) compared with the other yogurt formulations, indicating that the viability interfered with both pH and acidity. The maximum viability recorded was at ratio 1:3: (LacAT: *S. thermophilus)*, indicating that a supra-saturation with *Streptococcus* induces a decrease in the cell viability during storage (Figure 3). The results were similar to those obtained by Casarotti and colleagues [37], where the combination of *S. thermophilus* with *L. acidophilus* showed higher viability as opposed to *L. acidophilus* alone. The cell viability decline might be related to the secretion of inhibition metabolites (e.g., bacteriocins) produced by probiotics strains that might affect species of the same genus or related species. The broad inhibitory spectrum of UTNGt28 was previously investigated, demonstrating a high inhibitory capacity against Gram-positive and Gram-negative strains [17]. Complementary analysis of the UTNGt28 strain (T4) cell viability during storage obtained after initial fermentation at 35 °C, indicated that the cell counts were maintained relatively in the same amount, as UTNGt28 is a mesophilic strain, while, after incubation at 42 °C (the required temperature for *S. thermophilus*), the cell viability decreased considerably at 28 days (Supplementary Figure S4). The stability might be due to the initial conditions of fermentation, with a registered acidity of 0.61 (% lactic acid) at 35 °C and 0.67 (% lactic acid) at 42 °C. 

### 3.4. Protein and Fat Analysis in Probiotic Yogurt Formulations

During fermentation there are modifications of protein, sugars and lipids, giving pleasant changes to the flavor, aroma, texture and nutritional value of the final product. The fat content of the milk at the beginning of the experiment was 2.9%. Although the protein content in milk varied (in one batch was 2.9%), a regression analysis with the initial and final protein values indicated that the results were not affected, since there were no significant differences (*p* < 0.05). The yogurt formulations after 28 days of storage showed variations in the percentage of fat, with a decrease in the treatments that presented acidification (Table 4). According with the national norm [35], yogurt formulations that showed a minimum fat of 2.5% are considered whole fermented milk or whole yogurt, while the values from 1% to <2.5% are considered semi-skimmed fermented milk or semi-skimmed yogurt, thus, the formulations T1, T3, T7, T8 and T9 were classified as whole yogurt, while T2, T4 and T7 were classified as semi-skimmed yogurt. For the protein variable, all treatments are above the established limit of a minimum of 2.7%. The formulations T1 and T8 showed an increase of protein content compared with the other formulations and the whole milk used for the bacterial inoculation (Table 4). This might be due to the increase in the level of soluble proteins, free amino acids and non-protein nitrogen in the final product. Another study indicated that heat treatment of milk and the action of the initiating bacteria during the production of yogurt causes the breakdown of milk protein [38]. This implies that yogurt may be a better source of protein compared to yogurt drinks or other fermented milk drinks. The fat content registered in this research reduced considerably in the yogurt formulations with a lower pH after the fermentation, while minor changes were registered in the protein content, indicating that no degradation was evident after fermentation (Table 4). Previous research indicated a reduction in both fat and protein content in the final product after fermentation [39]. Based on our results, the fat decreased in all formulations depended on the bacterial combinations used in the fermentation process. Moreover, the bacteriological analysis indicated no coliforms in the yogurt formulations and the counted yeasts and molds were below the accepted values indicated by the national norm [35], thus all obtained formulations were satisfactory and acceptable in terms of quality. As the target strain UTNGt28 has antimicrobial activity towards coliforms [17], we suggest that this characteristic that will improve the quality of the yogurt, as the potential growth of incoming microorganisms during manipulation is inhibited 

**Figure 4:**
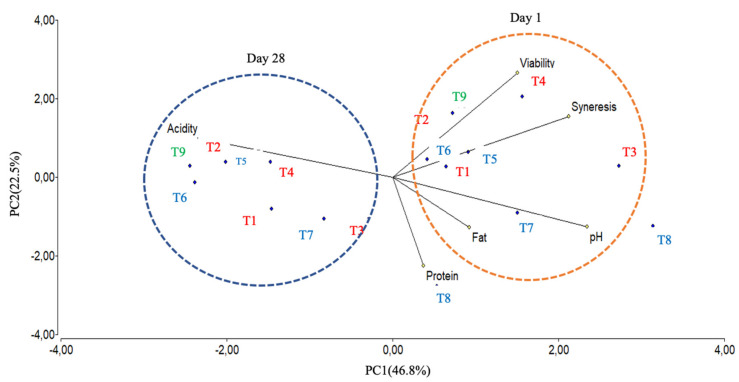
Principal component analysis of yogurt data at day 1 (red circle) and 28 of storage (blue circle). Legend: T1: UTNGt28 + *S. thermophilus* ATCC19258:1:1 (*g/g);* T2: UTNGt28 + *S. thermophilus* ATCC19258:1:3 (*g/g*); T3: UTNGt28 + *S. thermophilus* ATCC19258:3:1 (*g/g*); T4: UTNGt28; T5: LacAT+ *S. thermophilus* ATCC19258:1:1 (*g/g*); T6: LacAT+ *S. thermophilus* ATCC19258:1:3 (*g/g*); T7: LacAT+ *S. thermophilus* ATCC19258:3:1 (*g/g*); T8: LacAT; T9: *S. thermophilus* ATCC19258. The UTNGt28-based formulations are indicated with red font and the LacAT-based formulations with blue font; the commercial starter *S. thermophilus* ATCC19258 is indicated with green font, for better visualization.

A biplot was created using the PCA scores and factor loading to compare the similarities of the yogurt formulations obtained with the two bacteria and understand the relationship between the microbiological and chemical determinations. A PCA of the six factors demonstrates a clear separation between the yogurt formulations after one day and the end of storage (Figure 4). The PC 1 explained 46.8% of the total variance (day 28), while the PC 2 explained 22.5% (day 1). An additional component of 17.6% was accounted for by PC3 (data not shown). PC 1 was more heavily loaded in the negative (−) direction with titratable acidity (% lactic acid) and the PC2 in the positive (+) direction with the pH, cells viability, syneresis, fat and protein. The yogurt formulations at day 1 of storage varied considerably and were grouped mainly on their pH, viability (cells counts) and syneresis values. Nonetheless, the T4 and T9 formulations were found to exhibit similar behavior in terms of fermentation time, pH, acidity and fat. The T8 formulation displayed a distinct pattern, as it exhibited the highest and most constant pH value during storage compared with its corresponding formulations, as well as the lowest viability, indicating no probiotic capacity. We suggest that the drop of cell viability in T8 might be attributed to the increase in the concentration of lactic acid at the end of storage. 

## 4. Conclusions

Overall, the viability of probiotic bacteria in the new yogurt formulations depends upon the strain type and culture mixture ratio. The UTNGt28 (T4) has a similar behavior to the commercial *Streptococcus thermophilus* ATCC19258 (T9) with respect to the fermentation time, registered pH, and acidity during storage, indicating its promising capacity to be considered as an initiating culture. The yogurt formulations containing UTNGt28 maintained viable cell counts, while the formulation with *Lactococcus lactis* ATCC11454 only presented the greatest loss of cell viability during storage and does not fulfill the probiotic criteria regarding the cell survival during storage. This study presents relevant information on the physicochemical and microbial properties of yogurt formulations obtained with different mixtures of bacterial culture, which could guide the dairy industry in developing new probiotic products. Looking forward, the demand for probiotic yogurt is expected to increase in Ecuador, as consumers value healthy foods, and by definition, the yogurt market will likely be boosted, providing the opportunity to develop new products using native lactic bacteria species.

## Figures and Tables

**Table 1:** Pasteurized whole milk analysis.

Raw Milk	Values (This Study)	Standard Values *[23]
Acidity Titer (% lactic acid)	0.14–0.15	0.13–0.17
Fat (mass fraction %)	2.9	2.9–3
Relative Density at 20 ºC (g/mL)	1.029	1.029–1.033
Total protein (mass fraction %)	2.9–3.35	<2.9
pH	6.60–6.68	-

* The values represent the minimum and maximum accepted.

**Table 2:** Fermentation times to reach pH 4.5 for studied formulations for potential use in the manufacturing of yogurt.

Formulation	Bacterial Combination Ratio (*g/g*)	Time (h) *
T1	UTNGt28 + *S. thermophilus* ATCC19258: 1: 1	11 ± 0.10
T2	UTNGt28 + *S. thermophilus* ATCC19258: 1: 3	10 ± 0.08
T3	UTNGt28 + *S. thermophilus* ATCC19258: 3: 1	12 ± 0.02
T4	UTNGt28	9 ± 0.10
T5	LacAT + *S. thermophilus* ATCC19258: 1: 1	12 ± 0.25
T6	LacAT + *S. thermophilus* ATCC19258: 1: 3	10.5 ± 0.41
T7	LacAT + *S. thermophilus* ATCC19258: 3: 1	13 ± 0.09
T8	LacAT	14 ± 0.25
T9	*S. thermophilus* ATCC19258	8 ± 0.10

* The values represent means ± standard deviation (*n* = 3).

**Table 3:** Syneresis values of the yogurt formulations expressed as % of water retention.

Formulation	Bacterial Combination Ratio (*g/g*)	% of Water Retention (Day)	
		1	28
T1	UTNGt28 + S*. thermophilus* ATCC19258: 1: 1	51	43
T2	UTNGt28+ S*. thermophilus* ATCC19258: 1: 3	49	46
T3	UTNGt28+ S*. thermophilus* ATCC19258: 3: 1	55	41
T4	UTNGt28	53	42
T5	LacAT + S*. thermophilus* ATCC19258: 1: 1	53	41
T6	LacAT + S*. thermophilus* ATCC19258: 1: 3	48	38
T7	LacAT + S*. thermophilus* ATCC19258: 3: 1	48	41
T8	LacAT	51	41
T9	S*. thermophilus* ATCC19258	48	37

**Table 4:** Variation of fat and protein at day 28 of storage in the yogurt formulations.

Formulation	Bacterial Combination Ratio (g/g)	Fat Content (%)	Protein Content (%)	Product Characteristics Based on Fat Content
T1	UTNGt28 + S*. thermophilus* ATCC19258: 1: 1	2.5	3.38	whole yogurt
T2	UTNGt28 + S*. thermophilus* ATCC19258: 1: 3	2.4	3.18	semi-skimmed yogurt
T3	UTNGt28 + S*. thermophilus* ATCC19258: 3: 1	2.7	3.17	whole yogurt
T4	UTNGt28	2.4	3.05	semi-skimmed yogurt
T5	LacAT + S*. thermophilus* ATCC19258: 1: 1	2.4	3.13	semi-skimmed yogurt
T6	LacAT + S*. thermophilus* ATCC19258: 1: 3	2.3	3.35	semi-skimmed yogurt
T7	LacAT + S*. thermophilus* ATCC19258: 3: 1	2.5	3.21	whole yogurt
T8	LacAT	2.5	3.38	whole yogurt
T9	S*. thermophilus* ATCC19258	2.5	3.01	whole yogurt

## Supplementary Materials

The following are available online at https://www.mdpi.com/2076-2607/8/5/733/s1, Supplementary Figure S1: pH variation of yogurt formulations during storage with refrigeration. Supplementary Figure S2: Lactic acid (%) variation of yogurt formulations during storage with refrigeration. Supplementary Figure S3: Variation in pH and syneresis (%) during storage with refrigeration of yogurt containing UTNGt28 cells during storage after coagulation at 35 °C and 42 °C. Supplementary Figure S4: Cell counts of yogurt formulation containing UTNGt28 strain during storage after coagulation at 35 °C and 42 °C.
