# Supplementary material for: Probiotic Potential and Technological Properties of Bacteriocinogenic Lactococcus lactis Subsp. Lactis UTNGt28 from a Native Amazonian Fruit as a Yogurt Starter Culture
Source: Microorganisms. 2020 May 14;8(5):733. doi: 10.3390/microorganisms8050733 (PMC7285064; doi:10.3390/microorganisms8050733)
Supplement: Supplementary file 1 [file microorganisms-08-00733-s001.pdf]

microorganisms

MDPI

Supplementary Figures

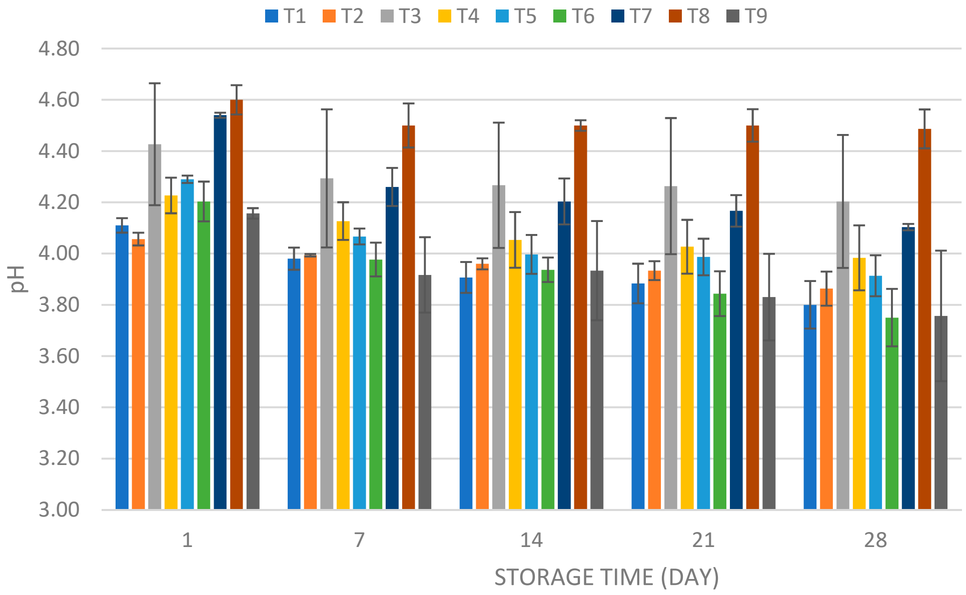

Figure S1. pH variation of yogurt formulations during storage with refrigeration. Legend: T1: UTNGt28 + *S. thermophilus* ATCC19258: 1: 1 (*g/g*); T2: UTNGt28 + *S. thermophilus* ATCC19258: 1: 3 (*g/g*); T3: UTNGt28 + *S. thermophilus* ATCC19258: 3: 1 (*g/g*); T4: UTNGt28; T5: LacAT+ *S. thermophilus* ATCC19258: 1: 1 (*g/g*); T6: LacAT+ *S. thermophilus* ATCC19258: 1: 3 (*g/g*); T7: LacAT+ *S. thermophilus* ATCC19258: 3: 1 (*g/g*); T8: LacAT; T9: *S. thermophilus* ATCC19258. The values represent the means ± standard deviation (n = 3).

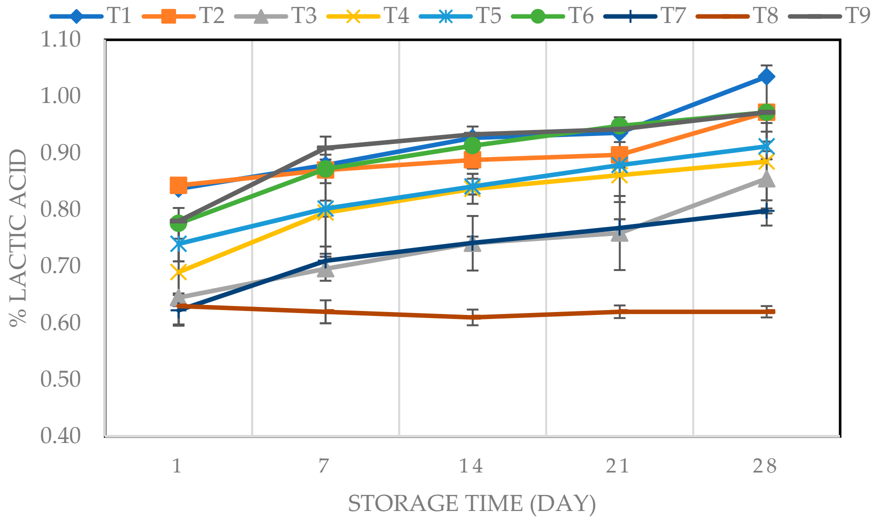

Figure S2. Lactic acid (%) variation of yogurt formulations during storage with refrigeration. Legend: T1: UTNGt28 + *S. thermophilus* ATCC19258: 1: 1 (*g*/*g*); T2: UTNGt28 + *S. thermophilus* ATCC19258: 1: 3 (*g*/*g*); T3: UTNGt28 + *S. thermophilus* ATCC19258: 3: 1 (*g*/*g*); T4: UTNGt28; T5: LacAT+ *S. thermophilus* ATCC19258: 1: 1 (*g*/*g*); T6: LacAT+ *S. thermophilus* ATCC19258: 1: 3 (*g*/*g*); T7: LacAT+ *S. thermophilus* ATCC19258: 3: 1 (*g*/*g*); T8: LacAT; T9: *S. thermophilus* ATCC19258. The values represent the means ± standard deviation (*n* =3).

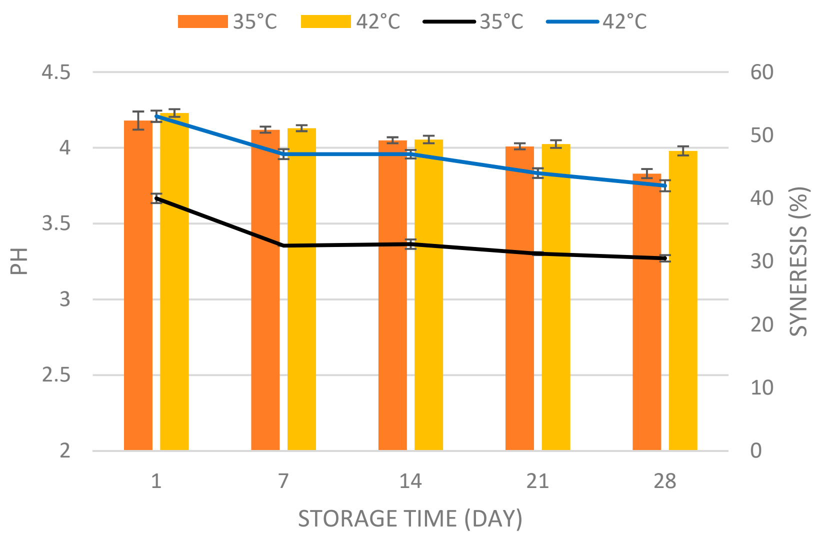

Figure S3. Variation in pH and syneresis (%) during storage with the refrigeration of yogurt containing UTNGt28 cells during storage after coagulation at 35 °C and 42 °C. The values represent the means ± SD (*n* = 3). The bars represent the pH values and the line represents the syneresis.

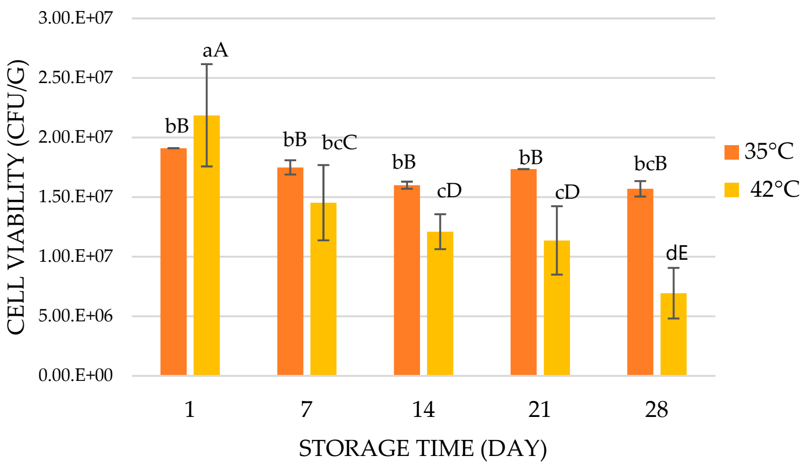

Figure S4. Cell counts of yogurt formulation containing UTNGt28 strain during storage after coagulation at 35 °C and 42 °C. The values represent the means ± standard deviation (n = 3). Values with different letters are significantly different ($p < 0.05$). Lower case letters show the difference between total cell counts-temperature (LSD with Bonferroni correction); Capital letters indicate the differences in the cell counts at different storage times (Duncan’s test).
